# RETRACTION: Hypobaric Hypoxia Imbalances Mitochondrial Dynamics in Rat Brain Hippocampus

**DOI:** 10.1155/nri/9860283

**Published:** 2026-02-14

**Authors:** Neurology Research International

RETRACTION: K. Jain, D. Prasad, S. B. Singh, E. Kohli, “Hypobaric Hypoxia Imbalances Mitochondrial Dynamics in Rat Brain Hippocampus,” *Neurology Research International*, 2015, 742059, https://doi.org/10.1155/2015/742059.

The above article, published online on 05 July 2015 in Wiley Online Library (https://wileyonlinelibrary.com), has been retracted by agreement between the journal Editorial Board Member, Jeff Bronstein, and John Wiley & Sons Ltd. The retraction has been agreed due to figure concerns, initially raised on PubPeer [1]. On further investigation, the below inconsistencies have been identified with Figure 4:

• In Figure 4b, there are unexpected repeated regions between the Mfn1/3dHH panel and the Mfn1/7dHH panel.
• In Figure 4b, the Mfn1/14dHH panel appears similar to the Mfn2/28dHH panel in Figure 4e.
• In Figure 4b, the Mfn2/7dHH panel appears similar to the Mfn1/21dHH panel in Figure 4e.
• In Figure 4b, the Mfn2/14dHH panel appears similar to the Mfn1/Control panel in Figure 4e.
• In Figure 4b, there are unexpected repeated regions between the Opa1/Control panel and the Fis1/28dHH panel.
• In Figure 4b, there are unexpected repeated regions between the Opa1/28dHH panel and the Fis1/3dHH panel.
• In Figure 4b, there are unexpected repeated regions between the Mff/3dHH panel and the Mff/7dHH panel.
• In Figure 4b, there are unexpected repeated regions between the Mff/14dHH panel and
  ◦ The Mff/3dHH panel in Figure 4e
  ◦ The Mff/21dHH panel in Figure 4e
• In Figure 4b, the p‐Drp1/control panel appears similar to the Mff/Control panel in Figure 4e when rotated to 270 degrees.
• In Figure 4b, there are unexpected repeated regions between the p‐Drp1/28dHH panel and the Mff/7dHH panel in Figure 4e.
• In Figure 4e, there are unexpected repeated regions between the Mff/3dHH panel and the Mff/28dHH panel.
• In Figure 4e, there are unexpected repeated regions between the Mff/21dHH panel and the Mff/28dHH panel
• In Figure 4e, there are unexpected repeated regions between the Mff/3dHH panel and the Mff/21dHH panel.

The author’s response was assessed by the editorial board who deemed the results now unreliable, and therefore, the article is retracted.

The authors disagree with the retraction.

## References

[1] A. biskrensis, “Hypobaric Hypoxia Imbalances Mitochondrial Dynamics in Rat Brain Hippocampus,” *PubPeer*, 2021, https://pubpeer.com/publications/240935003DD535A0A517BCED191030. 10.1155/2015/742059 PMC4509498 26236504

